# Estimates of the Nutritional Impact of Non-Participation in the National School Lunch Program during COVID-19 School Closures

**DOI:** 10.3390/nu14071387

**Published:** 2022-03-26

**Authors:** Amelie A. Hecht, Caroline Glagola Dunn, Eliza W. Kinsey, Margaret A. Read, Ronli Levi, Andrea S. Richardson, Erin R. Hager, Hilary K. Seligman

**Affiliations:** 1 Institute for Research on Poverty, University of Wisconsin-Madison, Madison, WI 53706, USA; 2 Department of Health Policy and Management, Harvard T.H. Chan School of Public Health, Boston, MA 02115, USA; glagocj@gmail.com; 3 Department of Family Medicine & Community Health, Perelman School of Medicine, University of Pennsylvania, Philadelphia, PA 19104, USA; eliza.kinsey@pennmedicine.upenn.edu; 4 Share Our Strength, No Kid Hungry, Washington, DC 20005, USA; mread@strength.org; 5 Department of Medicine, Division of General Internal Medicine, University of California, San Francisco, CA 94143, USA; ronli.levi@ucsf.edu (R.L.); hilary.seligman@ucsf.edu (H.K.S.); 6 Behavioral and Policy Sciences, RAND Corporation, Pittsburgh, PA 15213, USA; arichard@rand.org; 7 Department of Pediatrics, University of Maryland School of Medicine, Baltimore, MD 21201, USA; ehager@som.umaryland.edu; 8 Department of Epidemiology and Public Health, University of Maryland School of Medicine, Baltimore, MD 21201, USA

**Keywords:** school meal, school lunch, National School Lunch Program, COVID-19, obesity, disparities, diet quality, school closures

## Abstract

The COVID-19 pandemic resulted in widespread school closures, reducing access to school meals for millions of students previously participating in the US Department of Agriculture (USDA) National School Lunch Program (NSLP). School-prepared meals are, on average, more nutritious than home-prepared meals. In the absence of recent data measuring changes in children’s diets during the pandemic, this article aims to provide conservative, back-of-the-envelope estimates of the nutritional impacts of the pandemic for school-aged children in the United States. We used administrative data from the USDA on the number of NSLP lunches served in 2019 and 2020 and nationally representative data from the USDA School Nutrition and Meal Cost Study on the quality of school-prepared and home-prepared lunches. We estimate changes in lunchtime calories and nutrients consumed by NSLP participants from March to November 2020, compared to the same months in 2019. We estimate that an NSLP participant receiving no school meals would increase their caloric consumption by 640 calories per week and reduce their consumption of nutrients such as calcium and vitamin D. Because 27 to 78 million fewer lunches were served per week in March–November 2020 compared to the previous year, nationally, students may have consumed 3 to 10 billion additional calories per week. As students return to school, it is vital to increase school meal participation and update nutrition policies to address potentially widening nutrition disparities.

## 1. Introduction

School meals are a critical source of nutrition for millions of children and adolescents across the United States (US). Prior to the COVID-19 pandemic, the US Department of Agriculture (USDA) National School Lunch Program (NSLP) and School Breakfast Program served approximately 30 million lunches and 15 million breakfasts daily [1]. These programs provided up to half of students’ daily calories and reduced the prevalence of household food insecurity [2,3]. School meals are a particularly important strategy by which to promote equity among students from low-income families (three-quarters of participants) and Black and Hispanic students (who are more likely to participate than White and Asian students) [4,5]. Updated school nutrition standards in the Healthy, Hunger-Free Kids Act of 2010 made school meals more nutritious and lower in calories than meals brought from home [6,7,8]. School meals are thus critical in efforts to address socioeconomic and racial and ethnic disparities in nutrition and health outcomes.

The COVID-19 pandemic resulted in widespread school closures, forcing schools to rapidly innovate to maintain student access to emergency meals (i.e., meals served by schools during closures through programs such as grab-and-go pickup at centralized locations and home delivery) [9]. Despite extraordinary efforts, between March and November 2020 (excluding summer months), US school districts served approximately 45% fewer lunches than during the same period the year before [10]. Many of these lunches were likely replaced by lunches prepared at home, which are typically higher in calories and lower in critical nutrients [7,8]. Thus, school closures likely had substantial implications for the health of millions of US students.

The pandemic led to major changes in children’s daily lives, including restricted opportunities to leave the home due to lockdowns and school closures, changes in family income due to job loss and illness, and increased stress. These and other factors may have contributed to changes in children’s dietary behaviors. Several recent studies focused on diet quality among school-aged children in the US, Europe, Asia, and South America have found the pandemic was associated with changes in eating habits [11,12,13,14,15,16]. In a global systematic review that included 10 studies focused on dietary changes among children and adolescents during the first wave of pandemic lockdowns, the authors found that improved overall diet quality was detected in some studies, while worsened overall diet quality was detected in others [14]. Several studies included in the review identified increased consumption of unhealthy foods such as sweets, fried foods, and fast food among school-aged children.

In this article, we estimate how loss of school-prepared meals may have impacted caloric and nutrient intake among students from low-income US households and discuss evidence-based strategies to offset the potentially harmful effects of the pandemic. In the absence of recent data measuring changes in child diet quality during the pandemic, this article serves as a thought experiment, providing conservative, back-of-the-envelope estimates of the nutritional impacts of the pandemic that can help guide future research and practice.

## 2. Materials and Methods

We compared USDA meal reimbursement administrative data from March to November 2019 and 2020 to estimate the shortfall in the number of school-prepared lunches served [10]. Meal reimbursement administrative data include aggregate monthly counts of the number of meals served through the USDA meal programs and reported by state agencies. We estimated the caloric and nutrient impact of replacing zero, one, two, three, or four NSLP lunches per week with nutritionally equivalent emergency meals [10]. We assumed (1) emergency meals were of similar nutritional quality to NSLP meals, and (2) meals not replaced as emergency meals (ostensibly prepared at home) were of similar nutritional quality to meals that students had packed at home and brought to school prior to the pandemic (see Section 4.1 for a discussion of the limitations of these assumptions.) We used data from the USDA School Nutrition and Meal Cost Study (SNMCS), a nationally representative comparison of lunchtime dietary intake from students participating in the NSLP and matched nonparticipants in School Year 2014–2015 (Table 1 and Table A1) [7]. Detailed data collection and analysis methods are available in the SNMCS report [7]. Outcomes included change in kcals, macronutrients (including saturated fat and dietary fiber), and select micronutrients (vitamin D, calcium, iron, sodium, and potassium). We modeled changes in outcomes for an individual student and for the full population of students who received lunch through NSLP (based on the 2019 USDA Child Nutrition Tables data, 29.6 million lunches daily) [4]. We used basic arithmetic to produce back-of-the envelope estimates based on publicly available, nationally representative datasets.

All the data used in this analysis are available for public use and do not contain any personally identifying information. As this study did not meet the definition of human subjects research according to federal regulations (45 CFR 46), institutional review board approval was not required.

## 3. Results

On average, students who eat lunch prepared at home consume 128 additional calories compared to students who eat school-prepared lunch (Table 1). If, during the pandemic, a student ate no school-prepared lunches, caloric intake could increase by 640 calories per week (Table 2 and Table A2).

**Figure 1:**
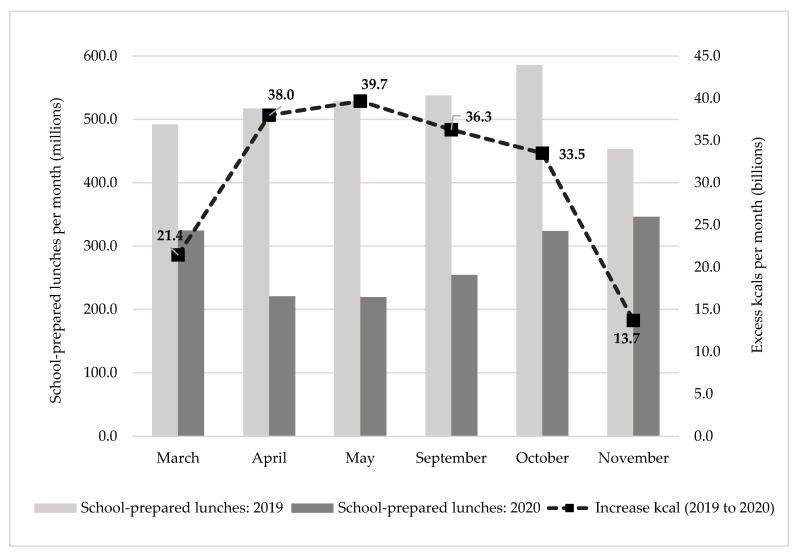
Total monthly lunchtime meals served and excess calories consumed by National School Lunch Program participants nationwide due to COVID related school closures, comparing March-November 2019 versus 2020. SOURCE: Data are the authors’ calculations based on lunchtime dietary recall data from the School Nutrition and Meal Cost Study, School Year 2014–2015 and the US Department of Agriculture November Keydata Report count of meals reimbursed monthly through the National School Lunch Program and Summer Food Service Program. Notes: Estimates assume National School Lunch Program lunches are replaced by emergency meals that meet National School Lunch Program standards, and that meals not replaced are equivalent in nutritional quality to home-prepared meals.

At the national level, 27 to 78 million fewer school-prepared lunches per week (or 107 to 310 million fewer school-prepared lunches per month) were served between March and November 2020 compared to the same period in 2019. (Figure 1) The gap narrowed as more schools reopened in fall 2020. This shortfall is associated with 3 to 10 billion additional calories consumed per week (or 14 to 40 billion additional calories consumed per month) among low-income US students. In May 2020, the month that saw the greatest shortfall of school-prepared lunches, students consumed 78 million fewer school-prepared lunches per week, which may have resulted in consumption of 10 billion more calories per week compared to during the same month the previous year.

Because school-prepared lunches differ in quality than those prepared at home, we also estimate an increase in consumption of some nutrients that dietary guidelines recommend be consumed in moderation (total fat, saturated fat, and sodium) and a decrease in the consumption of some nutrients that dietary guidelines recommend be consumed in greater quantities (calcium and vitamin D).

## 4. Discussion

Our back-of-the-envelope estimates indicate that the loss of school-prepared lunches during the pandemic may have resulted in considerable excess calorie consumption among students from low-income US households. Assuming 55% of lunches that would have otherwise been served in schools were replaced with emergency meals meeting NSLP standards, and the other 45% were replaced with home-prepared meals, excess weekly caloric consumption may have increased by up to 10 billion calories—the caloric equivalent of US children consuming nearly 41 million additional candy bars each week. The per-student daily increase of 128 calories associated with eating a home-prepared, rather than school-prepared lunch, if sustained, is likely sufficient to result in excess weight gain. Previous research suggests that 110–165 daily excess calories result in excess weight gain over a 10 year period among US children [17]. Recent evidence also suggests that children may have been less physically active and engaged in more sedentary behavior during school closures, which may further exacerbate concerns about unintended weight gain [16,18,19,20].

Approximately 18.5% of US children and adolescents are obese, and there are significant disparities by race, ethnicity, and socioeconomic status [21]. Childhood obesity is associated with adverse short- and long-term health outcomes, and higher risk of obesity and severe obesity in adulthood [22,23,24]. Unintended weight gain during the pandemic is an area of growing concern. A recent study found increased prevalence of child obesity since the start of the pandemic, with greater increases among children who are Black, Hispanic, and from households with low incomes [25].

Our findings also suggest that the pandemic-related loss of school lunches may have reduced the intake of key nutrients such as calcium and vitamin D, which are important for growth and development [26]. Those students most likely to participate in the NSLP—students who are Black, Hispanic, and from households with low incomes—already experience worse diet quality compared to their White and higher-income peers [27,28]. Thus, the pandemic may have widened disparities in intake of critical nutrients.

These models were designed to provide conservative estimates of how school closures may impact US student diet quality based on the best available evidence. There are currently no reliable national data that show the percent of schools that have taken advantage of USDA COVID-19 nutrition standard waivers, the nutritional quality of meals families have prepared at home since March 2020, or the percent of meals that have gone entirely unreplaced. These data will be critical to inform future research.

### 4.1. Limitations

Our analyses using SNMCS data provide a conservative estimate of changes in dietary intake due to school closures; however, our approach had some limitations. First, we assumed that emergency meals were of the same nutritional quality as NSLP meals. However, nationwide COVID waivers allowed schools to serve emergency meals of lower nutritional quality than those captured in SNMCS [9]. A case study of four large urban school districts during spring 2020 showed significant variation in the quality of meals served at emergency meal distribution sites [29]. The degree to which schools took advantage of these meal pattern waivers has not been systematically captured.

Second, we assumed that lunches prepared at home during the pandemic were of the same quality as lunches previously packed at home and brought to school. Evidence, however, suggests that students’ diet quality on weekends or during summer, when consuming lunches at home, is worse than both the quality of lunches prepared by schools or lunches packed at home and brought to school [30,31,32,33]. Families experiencing financial strain due to the pandemic may be serving meals at home that are of lower nutritional quality than those captured in SNMCS.

Third, we assumed that students who would have participated in NSLP on a typical school day had consistent access to meals at home during the pandemic. Prior studies show, however, that during school closures (e.g., summer vacation), food insecurity rises [34]. Therefore, some households may not have had the ability to replace these meals. The risk of food insecurity during the pandemic was not evenly distributed. For example, for students in rural areas, access to school meals may have been particularly reduced. Additionally, unemployment rates were disproportionately higher among non-White families, while unemployment benefits were disproportionately lower [35,36]. Resource-constrained households that experienced a loss of income may have had a harder time replacing school meals with nutritious and balanced meals at home and may have also faced challenges accessing emergency meal sites due to transportation barriers. While Pandemic-Electronic Benefits Transfer (P-EBT; monetary benefits to families of students who temporarily lost access to free or reduced-price school meals) undoubtedly helped alleviate this shortfall, the implementation of the P-EBT program was delayed and inconsistent across states, and program reach and adequacy has not been evaluated [37].

Fourth, estimates focus on lunch and do not account for breakfast, supper, or snacks [4]. Children may compensate for changes in calories consumed at lunch by consuming more or fewer calories at other mealtimes.

**Figure A1:**
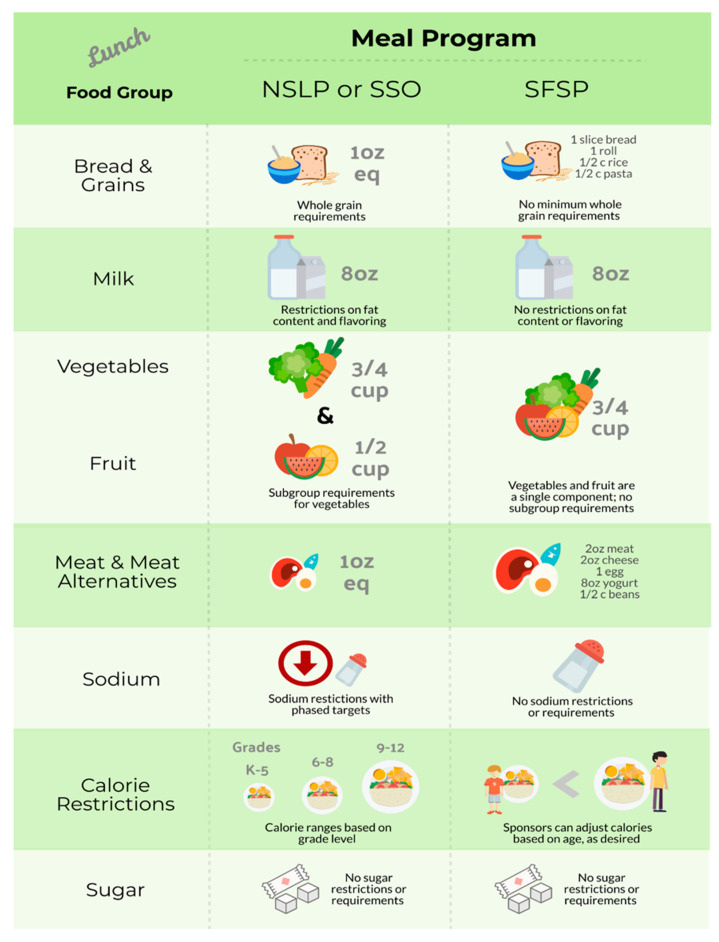
Meal pattern requirements ^†^ for meals served through the National School Lunch Program and Seamless Summer Option * compared to Summer Food Service Program. Adapted with permission from the Healthy Eating Research Strengthening the Impact of USDA’s Child Nutrition Summer Feeding Programs During and After the COVID-19 Pandemic brief [47]. Notes: * Sites serving meals using the Seamless Summer Option of the National School Lunch Program are traditionally required to adhere to the School Breakfast Program and National School Lunch Program meal pattern requirements. † Due to COVID, the US Department of Agriculture granted a nationwide waiver providing states the flexibility to serve meals that do not meet meal pattern requirements when needed [48].

Finally, NSLP meal guidelines differ by grade to reflect differences in nutritional needs between younger and older children. Meals served under pandemic waivers were not required to meet the same grade-specific nutrition standards [38]. For example, an emergency meal for an elementary and high school student likely looked the same, even though nutritional needs differ [26]. This approach may have helped alleviate hunger if households were able to stretch food, but may have resulted in overconsumption among younger children who were presented with larger-than-recommended portions (Figure A1).

These limitations contribute to imperfect projections; however, our conservative approach has, if anything, underestimated the negative impact of COVID on dietary intake. We do not use advanced statistical methods to calculate changes in diet quality, but rather aim to provide back-of-the-envelope estimates on which future analyses can build.

### 4.2. Policy Implications

Without policies to address the potential negative health impacts of long-lasting school closures, children may continue to grapple with the effects of weight gained during the pandemic even after schools reopen. Mitigating this damage may require stronger nutrition standards than are currently in effect for school meals and out-of-school meals. For example, during normal school closures (e.g., summer), many meals are distributed through the Summer Food Service Program (SFSP), which has lower nutrition standards than NSLP [39,40] (Figure A1). SFSP nutrition standards could be updated to align with the most recent dietary guidelines, with flexibilities for non-school sites, through Child Nutrition Reauthorization (CNR). CNR is the process by which Congress reauthorizes the federal child nutrition assistance programs and is expected to occur in 2022. Alternatively, higher reimbursement rates could be used to incentivize more schools to serve meals through the Seamless Summer Option (an alternative summer meal reimbursement option that many schools are using this school year owing to extended USDA COVID waivers, and which follows the same nutrition standards as NSLP). Further, strategies shown to increase school meal participation, such as the provision of universal free meals through the Community Eligibility Provision (CEP), could be widely adopted [41]. Other evidence-based policies that improve meal consumption include ensuring students have enough seated time during lunch and offering recess before lunch [42,43].

Learning from food distribution successes during the pandemic may streamline school meal access during future closures. Schools participating in CEP were able to transition particularly smoothly to distributing emergency meals at the beginning of the pandemic. For example, some CEP schools automatically began sending free meals to all students at home, and all students in CEP schools were eligible for P-EBT benefits [44]. Additionally, because CEP eliminates the need for schools to use general or other non-federal funds to cover student meal debt, CEP may help participating schools distribute meals during closures by strengthening school finances [45,46]. In the upcoming CNR, Congress could reauthorize CEP and expand it to more schools, increase meal reimbursement rates, and/or implement a national universal free meal program.

While this study focused on changes in diet quality among US children, emerging evidence suggests that school closures and lockdowns may have had similar negative effects on diet quality among children in other countries [11,12,13,14,15]. As such, policymakers and practitioners in other countries may also consider using strategies demonstrated to be effective in their local contexts to promote healthy dietary behaviors among children.

School meals play a critical role in students’ dietary quality, now more than ever. It is vital that we implement evidence-based school nutrition policies to offset potential harmful consequences of the pandemic.

## Figures and Tables

**Table 1:** Lunchtime dietary intake, comparing National School Lunch Program participants and matched nonparticipants on a typical school day.

	NSLP Participants	NSLP Nonparticipants	Difference (Nonparticipants–Participants)
kcal	515	643	+128 *
Nutrient			
Total fat (g)	17	24	+7 *
Saturated fat (g)	5	7	+2 *
Carbohydrate (g)	71	87	+16 *
Protein (g)	23	23	0.0
Vitamin D (mcg)	4.5	1.8	−2.7 *
Calcium (mg)	361	321	−40
Iron (mg)	3.3	4.1	+0.8 *
Dietary fiber (g)	6	6	0.0
Sodium (mg)	833	1057	+224 *
Potassium (mg)	772	730	−42

SOURCE: NSLP participant and matched nonparticipant lunchtime dietary recall data from the School Nutrition and Meal Cost Study, School Year 2014–2015. NOTES: NSLP: National School Lunch Program. Asterisk (*) denotes statistically significant difference between NSLP participants and matched nonparticipants in analyses conducted by report authors.

**Table 2:** Estimated per-student total weekly change in lunchtime calories and nutrients consumed, assuming 0–4 lunches are replaced through COVID emergency meal distribution.

NSLP Lunches Replaced Per Week by Emergency Meals	0	1	2	3	4
Lunches Per Week Prepared at Home	5	4	3	2	1
Change in kcal	640	512	384	256	128
Change in nutrients					
Total fat (g)	35	28	21	14	7
Saturated fat (g)	10	8	6	4	2
Carbohydrate (g)	80	64	48	32	16
Protein (g)	0.0	0.0	0.0	0.0	0.0
Vitamin D (mcg)	−13.5	−10.8	−8.1	−5.4	−2.7
Calcium (mg)	−200	−160	−120	−80	−40
Iron (mg)	4.0	3.2	2.4	1.6	0.8
Dietary fiber (g)	0.0	0.0	0.0	0.0	0.0
Sodium (mg)	1120	896	672	448	224
Potassium (mg)	−210	−168	−126	−84	−42

SOURCE: Data are the authors’ calculations based on lunchtime dietary recall data from the School Nutrition and Meal Cost Study, School Year 2014–2015. Notes: NSLP: National School Lunch Program. Estimates assume 0–4 weekly NSLP lunches are replaced by emergency meals that meet NSLP standards, and that meals not replaced are equivalent in nutritional quality to home-prepared meals.

## Data Availability

National School Lunch Program participant and matched nonparticipant lunchtime dietary recall data: the School Nutrition and Meal Cost Study, Volume 4 (accessed on 1 March 2022, https://fns-prod.azureedge.net/sites/default/files/resource-files/SNMCS-Volume4.pdf). US Department of Agriculture meal reimbursement data (accessed on 1 March 2022, https://www.fns.usda.gov/data/november-2020-keydata-report and https://www.fns.usda.gov/pd/child-nutrition-tables). Daily dietary intake recommendations from the 2015–2020 Dietary Guidelines for Americans, Appendix 7 (accessed on 1 March 2022, https://health.gov/sites/default/files/2019-09/2015-2020_Dietary_Guidelines.pdf).

## Appendix A

**Table A1:** Dietary intake at lunch, comparing National School Lunch Program participants and matched nonparticipants on a typical school day, by school level.

	Elementary School			Middle School			High School		
	NSLP Participant	Nonparticipant	Daily Recommended	NSLP Participant	Nonparticipant	Daily Recommended	NSLP Participant	Nonparticipant	Daily Recommended
kcal	489	587	1200–1600	501	546	1600–1800	588	713 *	1800–3200
Nutrient									
Total fat (% kcal) ^a^	26.8	29.4	25–35	29.8	31.6	25–35	29.2	32.7 *	25–35
Saturated fat (% kcal) ^a^	8.6	9.4	<10	8.9	9	<10	9	10	<10
Carbohydrate (g) ^b^	69	82	130	66	75	130	78	93 *	130
Protein (g) ^b^	22	20	19	23	20 *	34	27	27	46–52
Vitamin D (mcg) ^b^	5.1	1.1 *	15	4.3	2.1 *	15	3	2.4	15
Calcium (mg) ^b^	360	301	1000	322	268 *	1300	394	358	1300
Iron (mg) ^b^	3.1	3.8	10	3.1	3.4	8	3.7	4.4 *	11–15
Dietary fiber (g) ^c^	6	6	16.8–19.6	6	6	22.4–25.2	6	6	25.2–30.8
Sodium (mg) ^d^	770	908	1900	794	902	2200	1015	1263 *	2300
Potassium (mg) ^e^	764	649 *	3800	715	638	4500	837	846	4700

SOURCE: NSLP participant and matched nonparticipant lunchtime dietary recall data from the School Nutrition and Meal Cost Study. Daily dietary intake recommendations from the 2015–2020 Dietary Guidelines for Americans, Appendix 7. Notes: NSLP: National School Lunch Program Asterisk (*) denotes statistically significant difference between National School Lunch Program participants and matched nonparticipants. ^a^. Acceptable macronutrient distribution range, ^b^. recommended dietary allowance; ^c^. per 1000 kcal; ^d^. tolerable upper intake level; ^e^. adequate intake.

**Table A2:** Estimated total population-level change in weekly lunchtime calories and nutrients consumed by National School Lunch Program participants (n = 29.6 million) nationwide, assuming 0–4 lunches weekly are replaced through COVID emergency meal distribution sites serving meals that meet the National School Lunch Program standards.

NSLP Lunches Replaced Per Week by Emergency Meals	0	1	2	3	4
Lunches Per Week Prepared at Home	5	4	3	2	1
Change in kcal	18,944	15,155	11,366	7578	3789
Change in nutrients					
Total fat (g)	1036	829	622	414	207
Saturated fat (g)	296	237	178	118	59
Carbohydrate (g)	2368	1894	1421	947	474
Protein (g)	0	0	0	0	0
Vitamin D (mcg)	−400	−320	−240	−160	−80
Calcium (mg)	−5920	−4736	−3552	−2368	−1184
Iron (mg)	118	95	71	47	24
Dietary fiber (g)	0	0	0	0	0
Sodium (mg)	33,152	26,522	19,891	13,261	6630
Potassium (mg)	−6216	−4973	−3730	−2486	−1243

Source: The data are the authors’ calculations based on lunchtime dietary recall data from the School Nutrition and Meal Cost Study and the US Department of Agriculture Child Nutrition Tables reported number of meals reimbursed daily through the National School Lunch Program. Notes: NSLP: National School Lunch Program. Estimates assume 0–4 weekly lunches are replaced through COVID emergency meal distribution sites serving meals that meet the National School Lunch Program standards and that meals not replaced are equivalent in nutritional quality to home-prepared meals.
